# Personal

**Published:** 1893-03

**Authors:** 


### Personal.

The seventieth birthday of Louis Pasteur, of Paris, was recently the occasion of a national—better international—demonstration to this learned *savant*.

---

Professor Verneuil, one of the most prominent surgeons of France, and Professor of Surgery at the University of Paris, has resigned his professorship on account of ill health.

---

Dr. S. A. Dunham was elected Secretary of the Section on Anatomy, Physiology, and Pathology of the Buffalo Academy of Medicine, in place of Dr. Snow, resigned.

